# Platelet-Rich Plasma Induces Autophagy and Promotes Regeneration in Human Dental Pulp Cells

**DOI:** 10.3389/fbioe.2021.659742

**Published:** 2021-09-08

**Authors:** Hanxin Xu, Fen Xu, Jiajia Zhao, Caixia Zhou, Jiarong Liu

**Affiliations:** ^1^ Department of Stomatology, Union Hospital, Tongji Medical College, Huazhong University of Science and Technology, Wuhan, China; ^2^ School of Stomatology, Tongji Medical College, Huazhong University of Science and Technology, Wuhan, China; ^3^ Hubei Province Key Laboratory of Oral and Maxillofacial Development and Regeneration, Wuhan, China

**Keywords:** platelet-rich plasma, autophagy, osteogenic differentiation, human dental pulp cells, dental pulp regeneration

## Abstract

Regenerative endodontic procedures using autologous platelet-rich plasma (PRP) can improve the biologic outcome of treatment. However, its mechanism of action on improving pulp regeneration is not fully elucidated. Autophagy was recently shown to be related to tissue repair and osteogenesis. Therefore, the objective of this study was to investigate the effect of PRP in dental pulp regeneration and to elucidate the role of autophagy involved in this process. Human dental pulp cells (hDPCs) were isolated from healthy dental pulp and co-cultured with an increasing concentration of PRP. Cellular migration and proliferation were determined by scratch assay, transwell assay, and cell-counting kit 8 assay. Osteogenic differentiation was clarified by using alkaline phosphatase staining, alizarin red staining, and real-time polymerase chain reaction (RT-PCR) to measure the gene expression levels of alkaline phosphatase, collagen-1, osteocalcin, dentin matrix protein 1, and dentin sialophosphoprotein. Autophagic bodies were observed by transmission electron microscopy and the expression of autophagy marker light chain 3B (LC3B) was determined by immunofluorescence staining. The mRNA and protein expression level of LC3B and Beclin-1 were quantified by qRT-PCR and western blotting. The effect of PRP on cellular migration, proliferation, and osteogenic differentiation was further investigated in the milieu of autophagy activator, rapamycin, and inhibitor, 3-methyladenine. Results showed that PRP promoted cell migration, proliferation, and osteogenic differentiation. Autophagic bodies were strongly activated and the expression level of LC3B and Beclin-1 was significantly promoted by PRP. Autophagy inhibition suppressed PRP-induced hDPCs migration, proliferation, and osteogenic differentiation, whereas autophagy activator substantially augmented PRP-stimulated migration, proliferation, and differentiation. Taken together, these findings suggested that PRP could effectively promote regenerative potentials associated with autophagy.

## Introduction

If immature teeth have a diffuse pulp infection or periapical periodontitis with an open apex, apexification with calcium hydroxide or apical barrier with mineral trioxide aggregate was adapted according to the traditional methods (Bonte et al., 2015). However, neither method above could promote root development (Andreasen et al., 2002). Regenerative endodontic methods mainly including revascularization and regeneration could enable these roots to continue growing in width and length (Sun et al., 2011). Recent reports demonstrated that revitalization of these infected immature teeth using platelet-rich plasma (PRP) provided a desirable outcome (Sachdeva et al., 2015). PRP separated from whole blood has been described as platelet concentrates. When these platelets are activated, they could release amounts of growth factors. These growth factors are confirmed to accelerate the healing process and promote pulp repair (Altaii et al., 2017). PRP also presented benefits as a natural scaffold for cell proliferation, migration, and differentiation in pulp regeneration (Torabinejad and Faras, 2012; Bezgin et al., 2015).

Although numerous reports indicated that PRP provided a promising choice in regenerative endodontic procedures, the mechanism of action remains to be fully determined. Autophagy is an intracellular catabolic process of self-degradation of cellular components (Sotthibundhu et al., 2018). It plays an important role in cellular nutrition supply, functions maintaining, growth, and removing anomalous cellular components that are accumulated in aging (Mizushima and Levine, 2010). Several proteins, including microtubule-associated protein light chain 3 B (LC3B) and Beclin-1, have been implicated in the process of autophagy. LC3B is located in both the inner and outer membranes of autophagosomes. Autophagy increases the conversion of LC3B I into LC3B II, so the accumulation of LC3B II is the most widely used marker for autophagy. Beclin-1 is an autophagy-initiated protein and therefore increases in Beclin-1 also accompany activation of the process (Mizushima, 2018). Autophagy was reported to be involved in cell proliferation, differentiation, and migration (Yang et al., 2015) and supposed to have an essential role in tissue repair and regeneration (Xiao et al., 2013).

We hypothesize that autophagy might be involved in PRP-mediated dental pulp regeneration. Therefore, this study aimed to evaluate the effect of PRP on migration, proliferation, and osteogenic differentiation of human dental pulp cells (hDPCs). We also explored the effects of PRP in the autophagic activity of hDPCs and the role of autophagy in PRP-induced migration, proliferation, and differentiation.

## Materials and Methods

### hDPCs Isolation and Culture

Dental pulp tissues were collected from healthy caries-free premolars or third molars from 13-to-25-year-old patients. Informed consent was obtained from the patients and the protocol was approved by the Ethics Committee of Union Hospital, Tongji Medical College, Huazhong University of Science and Technology. Briefly, under the sterile condition, tooth surfaces were cleaned by 75% ethanol; debris and periodontal ligament were then cleaned off by a scalpel blade. The tooth was slowly cut along cemento-enamel junction and pulp tissue was gently separated from the crown and root. Pulp tissue was then cut into small pieces and digested in a solution containing 3 mg/ml collagenase type I (Invitrogen) and 4 mg/ml dispase (Roche) for 1 h at 37°C. Digested pieces were cultured in DMEM supplemented with 20% FBS (Fetal Bovine Serum) and 1% penicillin-streptomycin (Huang et al., 2008; Karamzadeh et al., 2012). Cells were passaged three to four times and used in subsequent experiments.

### PRP Preparation and Activation

Human whole blood samples were harvested with informed consent from 6 (mean age 20 ± 15 years) healthy volunteer donors. The blood sample collection was also approved by the Institutional Research Ethics Committee of Union Hospital. PRP was separated from the blood samples by secondary centrifugation. The blood collection was firstly centrifugalized at 550 g for 10 min; the top plasma and middle platelet layers were then carefully transferred to a new tube. After secondly centrifugalizing the blood collection at 1,230 g for 15 min, the upper layer was discarded carefully and 1 ml of PRP containing precipitated platelets was obtained. Before each use, calcium chloride was added to PRP at a ratio of 1:9, and thrombin was added at 500 IU per 1 ml volume of PRP (Marx et al., 1998; Mizushima, 2018).

### Cell Migration Assay

Cell migration was firstly assessed by scratch assay. In short, hDPCs (7 × 10^5^) were seeded into 6-well plates and incubated for 24 h. The cells were scored with a sterile pipette tip to leave a plus shape scratch of approximately 0.4–0.5 mm in width. The culture medium was then changed into DMEM containing 1 and 10% FBS and 1, 5, 10, and 20% PRP separately in different groups. The cell migration was observed with an inverted microscope at 24 h.

In transwell assay, hDPCs (6 × 10^4^) were suspended in 200 uL of serum-free DMEM and were added to the upper chamber of the insert (transwell plates are 6.5 mm in diameter with 8 µm pore filters; Corning Costar, Cambridge, MA). The culture medium in the lower chamber was changed into 500 uL of DMEM with 1 and 10% FBS and 1, 5, 10, and 20% PRP, respectively. After 24 h, the upper chambers were washed with PBS and the cells inside the chamber were gently wiped off by a cotton swab. The cells beneath the filter were fixed with 4% paraformaldehyde and then stained with crystal violet for 30 min. The number of the migration cells was observed and counted under an inverted microscope. All experiments were done in triplicate.

### Proliferation Assay

The effect of PRP on the proliferation of hDPCs was evaluated by using a cell-counting kit 8 assay (CCK-8, Dojindo, Japan). Briefly, hDPCs (3 × 10^3^) were seeded into 96-well plates and divided into six groups. After overnight incubation, the medium was changed into different concentrations of FBS (1 and 10%) and PRP (1, 5, 10, and 20%) respectively. At the indicated time points (1, 3, 5 days), cells were quantified using CCK-8 assay and absorbance was measured at 450 nm in quintuplicate wells per condition.

To further explore the effect of autophagy on cell migration and proliferation in PRP-treated hDPCs, autophagy modulation was employed. Cells were pre-treated with rapamycin (RAP) (100 nM, MCE, China) or 3-methyladenine (3-MA) (5 mM, MCE, China) before incubation with 5% PRP in scratch assay, transwell assay, and CCK-8 assay.

### Immunofluorescence Staining

For immunofluorescence detection, hDPCs were seeded on top of coverslips in 12-well plates. After overnight incubation, the culture medium was changed into DMEM with 1 and 10% FBS and 1, 5, 10, and 20% PRP separately in different groups. After incubation for 24h, the cells were fixed in 4% paraformaldehyde, permeabilized with 0.5% Triton X-100, and blocked with 1% BSA. LC3B (1:500, Abclone, China) was added as primary antibody and incubated overnight at 4°C. Alexa Fluor 488 (Proteintech) green fluorescence conjugate goat anti-mouse was used as a secondary antibody (1:200). Alexa Fluor cy3 phalloidin red fluorescence conjugate (1:500) was then applied to mark the actin cytoskeleton, and subsequently, the DPSCs were treated with 4′, 6-diamidino-2-phenylindole (DAPI, 1:1,000, Biosharp, China). The coverslips were mounted on a microscope slide with an embedding medium and observed under a laser scanning confocal microscope. To investigate the effect of autophagy on immunofluorescence, cells were cultured in the presence of an inhibitor or agonist of autophagy.

### Transmission Electron Microscopy

hDPCs (5 × 10^6^) were cultured with 10% FBS or 5% PRP for 24 h, digested with 2.5% trypsin/EDTA (HyClone, America), centrifuged, and fixed with 2.5% glutaraldehyde (Sigma) overnight at 4°C. The samples were examined using a transmission electron microscope (Hitachi HT7700-SS, Hitachi, Japan) operating at 75 kV.

### Alkaline Phosphatase (ALP) Staining

hDPCs were cultivated in mineralized medium containing 10% FBS and 1, 5, 10, and 20% PRP for 7 days and fixed with 4% paraformaldehyde. The cells were stained with an ALP assay kit (Jiancheng Bioengineering Institute, China) according to the manufacturer’s instructions. To investigate the role of autophagy in PRP-induced cell osteogenic differentiation, autophagy inhibitor or enhancer was also introduced.

### Alizarin Red Staining

hDPCs (4 × 10^4^) were seeded into 24-well culture plates and cultured in osteogenic induction medium (Cyagen, China). The cells were divided into five groups and in each group 10% FBS and 1, 5, 10, and 20% PRP were added, respectively. Cultures were stained with Alizarin Red (Servicebio, China) on day 21 to show deposits of calcium phosphate according to the manufacturer’s instructions. Shortly, the cells were fixed with 4% paraformaldehyde for 30 min and stained by the Alizarin Red (Servicebio, China) for 15 min.

### RNA Isolation and Quantitative Real-Time Polymerase Chain Reaction (qRT-PCR)

Total RNA was isolated from hDPCs after 3 weeks of incubation (LC3B, Beclin-1 for 24 h, ALP for 7 days) and reversely transcribed into complementary DNA using the RNA Isolation Total RNA Extraction Reagent (Vazyme, China) and HiScript III RT SuperMix for qPCR (Vazyme, China). qRT-PCR was performed in triplicate using an AceQ Universal SYBR qPCR Master Mix (Vazyme, China). The reaction conditions were as follows: polymerase activation at 95°C for 10 min, 40 cycles of denaturation at 95°C for 15 s, and annealing and extension at 60°C for 1 min. The primers for the reference gene *β*-actin and for the target genes ALP, collagen-1(COL 1), osteocalcin (OCN), dentin matrix protein 1 (DMP-1), dentin sialophosphoprotein (DSPP), LC3B, and Beclin-1 are shown in Table 1.

**TABLE 1:** The primers for the reference genes.

Gene name	Forward (5’-3’)	Reverse (5’-3’)
GAPDH	ATT​CCA​TGG​CAC​CGT​CAA​GG	TCG​CCC​CAC​TTG​ATT​TTG​GA
ALP	TGA​GAG​TGA​CGA​GAA​AGC​CAG​G	TTC​CGT​GCG​GTT​CCA​GAT​GAA
OCN	TCA​CAC​TCC​TCG​CCC​TAT​TG	TGC​TTG​GAC​ACA​AAG​GCT​G
COL-1	TAC​CGG​GCT​GAT​GAT​GCC​AAT	ATC​TTG​AGG​TCA​CGG​CAG​GT
DSPP	GGG​CCA​TTC​CAG​TTC​CTC​AAA	TTC​ATG​CAC​CAG​GAC​ACC​ACT
DMP-1	ATC​CTG​TGC​TCT​CCC​AGT​AAC​C	ATG​ACT​CAC​TGC​TCT​CCA​AGG​G
Beclin-1	CCA​TGC​AGG​TGA​GCT​TCG​T	GAA​TCT​GCG​AGA​GAC​ACC​ATC
LC3B	GAT​GTC​CGA​CTT​ATT​CGA​GAG​C	TTG​AGC​TGT​AAG​CGC​CTT​CTA

### Western Blot Analysis

hDPCs were cultured in 10% PBS, 5%PRP, RAP, or 5%3-MA for 24 h; protein was extracted using RIPA buffer with fresh protease inhibitors (InvivoGen). Protein concentration was measured by the BCA kit (Beyotime). The proteins were separated by electrophoresis on an SDS-PAGE gel and then transferred onto a PVDF membrane. After blocking for 1 h with skim milk, the membranes were incubated with primary antibodies against LC3B (1:1,000; Abclonal), Beclin-1 (1:1,000; Abclonal), and GAPDH (1:1,0000; Proteintech) overnight at 4°C. Then, the membranes were incubated with secondary antibodies for more than 1 h. The protein-antibody complexes were then visualized using the enhanced chemiluminescence detection system.

### Statistical Analysis

Data from different experiments were represented as mean ± standard deviation and analyzed by SPSS 20.0. Student’s *t*-test or one-way ANOVA was used to analyze the significant difference. *p* < 0.05 was considered statistically significant.

## Results

### Cell Migration and Proliferation of hDPCs

**FIGURE 1:**
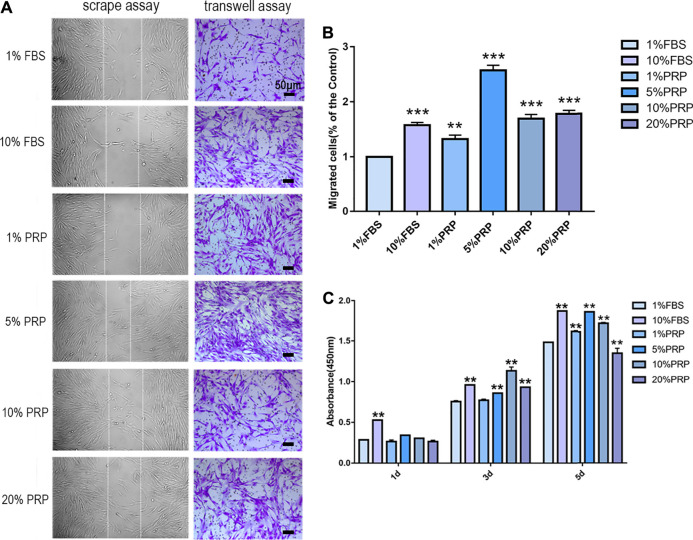
PRP enhanced cell migration and proliferation of hDPCs. **(A)** In scrape assay, hDPCs were seeded into 6-well plates in the presence of 1 and 10% FBS and 1, 5, 10, and 20% PRP for 24 h. In transwell assay, hDPCs were seeded into the upper chamber of transwell plates, with different concentrations of FBS or PRP in the lower chamber for 24 h (scale bar, 50 μm). **(B)** Measurement of the number of migrated hDPCs in a transwell assay. **(C)** The proliferation of hDPCs was assessed by a CCK-8 assay. Data in **(B)** and **(C)** are representative of three independent experiments. Data are presented as mean ± standard deviation. Significantly different groups, *p < 0.05, **p < 0.001.

In scrape assay, the results showed that 5, 10, and 20% PRP significantly promoted cell migration of hDPCs (Figure 1A) and 5% PRP showed the best effect. Simultaneously, in the transwell assay (Figures 1A,B), the number of cells that migrated toward 10% FBS and 1, 5, 10, and 20% PRP was significantly higher than the control group (1% FBS) (Figures 1A,B). The number of migrating cells toward 5% PRP was also the highest (Figure 1B).

To examine the effect of PRP on cell proliferation, a CCK-8 test was performed. The results demonstrated that there were no significant differences in cell proliferation rates between groups on day 1. On day 3, PRP at 5, 10, and 20% concentration significantly increased cell proliferation rates. On day 5, the cell cultured in 1, 5, and 10% PRP showed significantly higher proliferation rates, while a significant reduction in cell proliferation was observed with 20% PRP concentration than the control group (1% FBS) (Figure 1C).

### Osteogenic Differentiation of hDPCs

**FIGURE 2:**
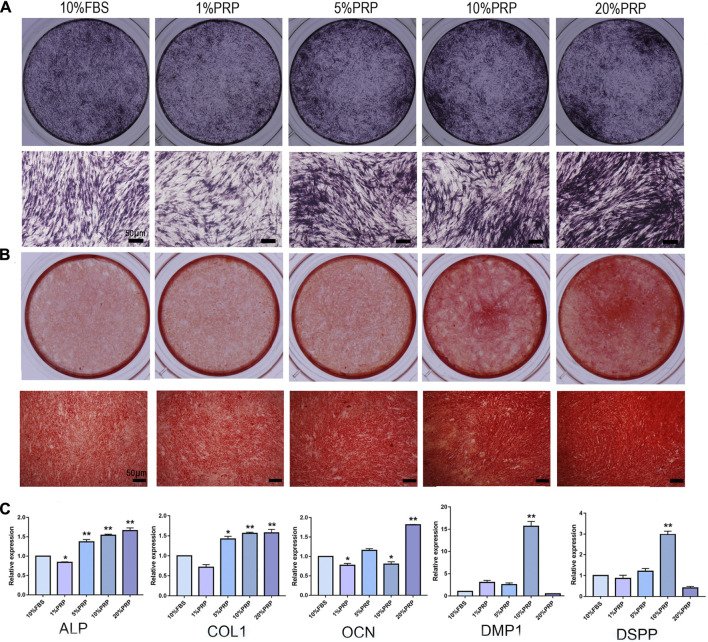
Effect of PRP on cell osteogenic differentiation of hDPCs was assessed by ALP, alizarin red staining, and real-time quantitative PCR. **(A)** Cells cultured in the presence of different concentrations of PRP were fixed and stained for ALP (scale bar, 50 μm). **(B)** Cells were fixed and stained with alizarin red (scale bar, 50 μm). **(C)** Real-time quantitative-PCR analysis of ALP, COL 1, OCN, DMP-1, DSPP mRNA expression in different groups. Data in **(C)** are representative of five independent experiments. Data are presented as mean ± standard deviation. Significantly different groups, *p < 0.05, **p < 0.001.

To explore the effects of PRP on osteogenic differentiation, ALP and alizarin red staining were analyzed. The hDPCs cultured in 5, 10, and 20% PRP increased the level of ALP staining than the control group (10% FBS) (Figure 2A). The alizarin red results showed that PRP increased mineralized nodule formation (5, 10, and 20%) (Figure 2B). The group treated with 20% PRP demonstrated the highest osteogenic induction capacities.

To determine the transcriptomic changes accompanied by PRP treatment, we performed qRT-PCR. ALP, Col-1, OCN, DMP-1, and DSPP are important genes for osteogenic differentiation. The expression levels of ALP and Col-1 were highly elevated in 5, 10, and 20% PRP-treated group (Figure 2C). The expression level of OCN was dramatically increased in the 20% PRP group, while DSPP and DMP-1 expression were significantly induced by 10% PRP (Figure 2C).

### Autophagy Activation by PRP

**FIGURE 3:**
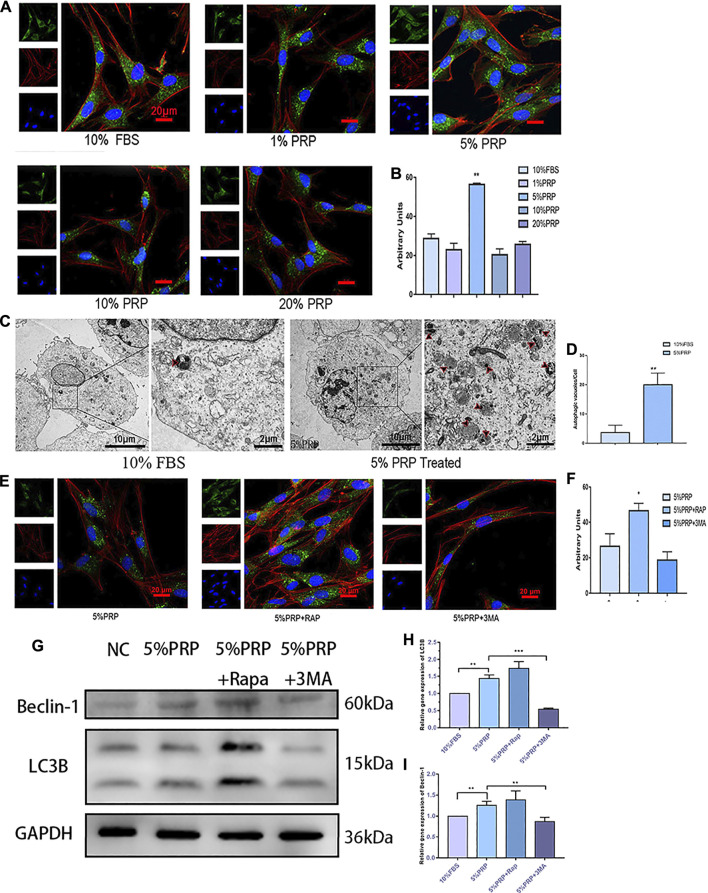
**(A)** Autophagy activation by PRP. By immunofluorescence staining, 5% PRP obviously increased the expression of LC3B (scale bar, 20 μm). **(B)** The graph shows the quantitative analysis of LC3B fluorescence by Image J. **(C)** By transmission electron microscope, 5% PRP-treated hDPCs showed a large number of autophagic vacuoles. **(D)** The number of autophagic vacuoles per cell. Experiments were repeated three times. **(E)** Immunofluorescence staining of autophagy-associated protein was elevated by rapamycin, whereas it was reduced by 3-MA (scale bar, 20 μm). **(F)** Quantitative analysis of LC3B fluorescence was estimated by Image J. **(G)** Western blot analysis of the autophagy-associated proteins levels in the hDPCs treated with 5% PRP, rapamycin (RAP) (100 nM) + 5% PRP, or 3-methyladenine (3-MA) (5 mM) + 5% PRP for 24 h. **(H)** LC3B and Beclin-1**(I)** mRNA expression were analyzed by quantitative PCR. Data in **(B)**, **(C)**, **(D)**, **(F)**, **(H),** and **(I)** are presented as mean ± standard deviation. Significantly different groups, *p < 0.05, **p < 0.001.

To investigate the activation of autophagy by PRP treatment, immunofluorescence staining was firstly employed to detect the expression of the autophagy-associated protein (LC3B). As shown in Figures 3A,B, the expression of LC3B was increased significantly by PRP at 5% concentration. The results of the transmission electron microscope showed that a large number of autophagic vacuoles (arrows in Figure 3C) were observed in hDPCs incubated with 5% PRP (Figure 3D). Consistent with the immunofluorescence staining results, the western blotting and qRT-PCR results showed that expression of autophagy-associated proteins, LC3B and Beclin-1, were increased by 5% PRP (Figures 3G–I). The result suggested that autophagy was strongly activated by PRP treatment.

After the agonist and inhibitor of autophagy were added, the immunofluorescence staining of LC3B was increased in the presence of rapamycin and decreased by 3-MA (Figures 3E,F). The expression level of autophagy-related genes and protein expressions, LC3B and Beclin-1, was also elevated by rapamycin and reduced by 3-MA (Figures 3G–I).

**FIGURE 4:**
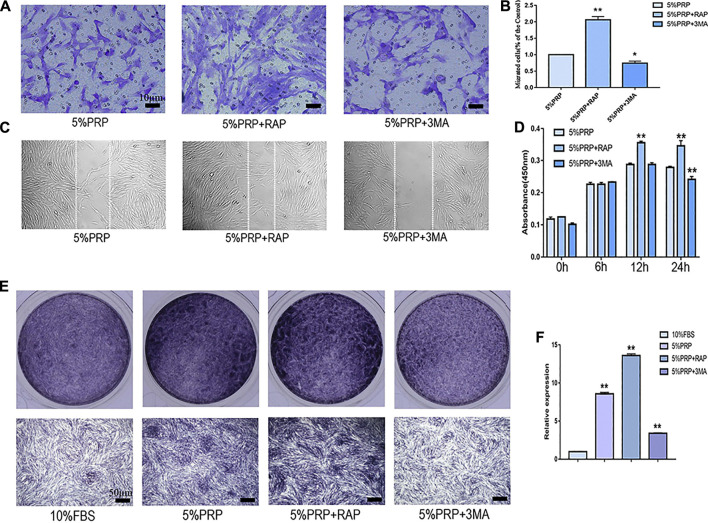
Cell migration, proliferation, and osteogenic differentiation were suppressed by autophagy inhibitor and promoted by autophagy agonist. hDPCs were treated with 5% PRP, rapamycin (RAP) (100 nM) + 5% PRP, or 3-methyladenine (3-MA) (5 mM) + 5% PRP. **(A)** By transwell assay, representative images of the migrated cells in different groups (scale bar, 10 μm). **(B)** The graph shows the number of migrated cells in different groups by transwell assay. **(C)** Representative images of the migrated cells were evaluated by scrape assay. **(D)** The CCK-8 assay was used to assess the proliferation ratio up to 24 h. **(E)** ALP staining of the four groups on day 7 (scale bar, 50 μm). **(F)** ALP mRNA expression was analyzed by quantitative PCR. Experiments were repeated three times. Data in **(B), (D), (E)** are presented as mean ± standard deviation. Significantly different groups, *p < 0.05, **p < 0.001.

In the presence of an autophagy activator (RAP), cell migration was promoted in both transwell (Figures 4A,B) and scratch assay (Figure 4C). In the presence of inhibitor (3-MA), the migration of hDPCs was weakened in both scrape and transwell assay (Figures 4A–C). In the CCK-8 assay, the proliferation of hDPCs was enhanced after treatment with autophagy activator at 12 and 24 h time points. Cell proliferation was weakened in the presence of autophagy inhibitor at 24 h (Figure 4D). The ALP staining and quantitative-PCR results revealed that autophagy inhibition suppressed PRP-induced hDPCs osteogenic differentiation, whereas autophagy activator augmented PRP-stimulated differentiation (Figures 4E,F). These data suggested that autophagy was involved in cell migration, proliferation, and differentiation induced by PRP.

## Discussion

Endodontic treatments are widely used in clinical work (Ng et al., 2007; 2008). However, for incompletely developed teeth, conventional root canal therapy would have a poor prognosis (Harlamb, 2016). On one hand, these teeth often have open and divergent apices, and it is difficult to clean and obturate these roots with traditional techniques and materials. On the other hand, the dentinal walls of these teeth are very thin and weak, which makes them prone to fracture with stress-overload (Lawley et al., 2004). The regenerative endodontic procedure can regenerate pulp-like tissue and promote root development of these teeth (Sachdeva et al., 2015; Kim et al., 2018; Staffoli et al., 2019). PRP contains many growth factors and provides desirable outcomes for the revitalization of immature teeth (Li et al., 2014; Diogenes and Ruparel, 2017). Therefore, there is wide interest in the PRP-mediated regeneration of dental pulp (Otero et al., 2017; Chai et al., 2019).

Dentinal regeneration requires the involvement of several physiological processes in hDPCs, including cell migration, proliferation, and osteogenic differentiation (Yeom et al., 2016; Zhang et al., 2017). The present study investigated the regenerative activity of hDPCs cultured with different concentrations of PRP and we demonstrated that PRP showed significant regenerative potential. We firstly demonstrated that cell migration and proliferation were significantly induced by PRP. The osteogenic experiments showed that PRP induced significantly greater mineralization of hDPCs. Some recent studies have developed culture conditions for in vitro expansion of cells treated by PRP (Lucarelli et al., 2003; Yeom et al., 2016; Pandey et al., 2019; Hahn et al., 2020). These research articles also focused on optimizing concentrations of PRP used for treatments (Lucarelli et al., 2003; Pandey et al., 2019; Wang et al., 2019; Hahn et al., 2020). Pandey et al. reported that 2.5–20% PRP (v/v) concentrations promoted cell migration and proliferation *in vitro*, while 40% PRP suppressed their migration and proliferation (Pandey et al., 2019). Another study demonstrated that PRP at 1–5% (v/v) concentration induced cell proliferation, while PRP at 30–100% concentrations suppressed cell proliferation (Choi et al., 2005). PRP with different concentrations was also found to have different capacities for MSC osteogenesis (Wang et al., 2019). Compared with the previously published results, we observed that PRP concentrations with up to 20% (v/v) could promote cell migration of hDPCs, with a PRP concentration of 5% to be the most optimal. Our data also demonstrated that PRP concentrations with up to 10% stimulated proliferation of hDPCs *in vitro*, while a significant reduction in cell proliferation was observed with 20% PRP concentration at day 5. Our osteogenic differentiation results showed that the osteogenic mineralization of hDPCs was stimulated by PRP ranging from 5 to 20%, with a PRP concentration of 20% to be the most optimal. The reported optimal concentrations for cell migration, proliferation, and differentiation varied between studies. These differences can be attributed to a variety of preparation procedures and methodology of PRP (Pandey et al., 2019). Furthermore, the difference can also be affected by the health status difference and personal condition of donors (Pandey et al., 2019). In our observations, PRP with up to 20% (v/v) concentration was beneficial for the regenerative potential of hDPCs.

However, to the best of our knowledge, we still have limited information on the action mechanism of PRP. Autophagy is an intracellular catabolic process by which protein aggregates and the damaged organelles are degraded. Autophagy was reported to have been involved in various cell procedures, such as cell migration, osteogenic differentiation of MSC, and odontoblast differentiation (Pantovic et al., 2013; Pei et al., 2016). To our knowledge, no study was done to evaluate the role of autophagy in PRP-induced pulp regeneration. Several studies have been published about the involvement of autophagy in the process of PRP treatment. Moussa et al. demonstrated that PRP increased significantly the cell proliferation of chondrocytes and promoted autophagosome and cartilage formation in osteoarthritic chondrocytes (Moussa et al., 2017). While another study demonstrated that PRP inhibited autophagic pathways and highlighted the regenerative effects against induced arthritis (Shafik et al., 2019).

In the present study, we hypothesized that autophagy might be involved in PRP-mediated cell migration, proliferation, and osteogenic differentiation in hDPCs. We firstly demonstrated that the expression of autophagy marker (including LC3B and Beclin-1) was increased significantly in 5% PRP-treated hDPCs. We also observed increased autophagosome formation by transmission electron microscope. These results suggested that autophagy was induced by PRP. In the following study, we proved that cell migration, proliferation, and osteogenic differentiation were upregulated in the presence of autophagy activator and downregulated in the presence of inhibitor. These results indicated that autophagy might be a crucial contributor to the regenerative ability of PRP. Former studies showed that autophagy was observed during odontoblast differentiation (Wang et al., 2017; Zhang and Chen, 2018) and even more in cells with LPS stimulation (Pei et al., 2016). These results suggested that autophagy modulated the odontoblast differentiation. Our results also demonstrated that PRP treatment could effectively promote regenerative potential associated with autophagy. Further studies are required to understand the molecular mechanism in PRP-mediated regeneration.

However, we have several limitations in the study. Firstly, the experiment was conducted in cultured hDPCs, and we speculate that, *in vivo*, the reaction of the cells to PRP stimulation might be more complex and varied compared with that in *in vitro* environment. So, further studies were required like a study in an animal model and we hope that it could provide new insights into the biology of PRP in dental pulp regeneration. Secondly, platelet and growth factor levels in PRP varied greatly due to individual differences in the donor and this may influence the research results. Thirdly, the autophagy inhibitory drug 3-MA was used in this study. 3-Methyladenine was a widely used autophagy inhibitor and elicited a significant reduction in cell viability (Chicote et al., 2020). According to Chicote J et al., the cytotoxicity induced by 3-MA correlated with massive DNA damage in many culture cell lines. We measured the effects of DNA damage by 3-MA in hDPCs and our results showed that 5 mM were non-cytotoxic concentrations of 3-MA for hDPCs (Supplementary Figure S1). Even though, 3-MA is called for cautionary usage in the future study.

In conclusion, our study suggested cell migration, proliferation, and osteogenic differentiation of hDPCs were promoted by PRP ranging from 5 to 20% concentration. Autophagy was triggered by PRP and might be a crucial contributor to the regenerative ability of PRP. This study will provide useful therapeutic strategies for the application of PRP in dental pulp regeneration.

## Data Availability

The original contributions presented in the study are included in the article/Supplementary Material; further inquiries can be directed to the corresponding author.
