# Supplementary material for: Platelet-Rich Plasma Induces Autophagy and Promotes Regeneration in Human Dental Pulp Cells
Source: Front Bioeng Biotechnol. 2021 Sep 8;9:659742. doi: 10.3389/fbioe.2021.659742 (PMC8455824; doi:10.3389/fbioe.2021.659742)
Supplement: Supplementary file 1 [file Table1.DOC]

Table 1.The primers for the reference genes

| Gene name | Forward(5'-3') | Reverse(5'-3') |
| --- | --- | --- |
| GAPDH | ATTCCATGGCACCGTCAAGG | TCGCCCCACTTGATTTTGGA |
| ALP | TGAGAGTGACGAGAAAGCCAGG | TTCCGTGCGGTTCCAGATGAA |
| OCN | TCACACTCCTCGCCCTATTG | TGCTTGGACACAAAGGCTG |
| COL-1 | TACCGGGCTGATGATGCCAAT | ATCTTGAGGTCACGGCAGGT |
| DSPP | GGGCCATTCCAGTTCCTCAAA | TTCATGCACCAGGACACCACT |
| DMP-1 | ATCCTGTGCTCTCCCAGTAACC | ATGACTCACTGCTCTCCAAGGG |
| Beclin-1 | CCATGCAGGTGAGCTTCGT | GAATCTGCGAGAGACACCATC |
| LC3B | GATGTCCGACTTATTCGAGAGC | TTGAGCTGTAAGCGCCTTCTA |
