# Supplementary material for: Platelet-Rich Plasma Induces Autophagy and Promotes Regeneration in Human Dental Pulp Cells
Source: Front Bioeng Biotechnol. 2021 Sep 8;9:659742. doi: 10.3389/fbioe.2021.659742 (PMC8455824; doi:10.3389/fbioe.2021.659742)
Supplement: Supplementary file 2 [file Table2.DOCX]

***Supplementary Figure***

3-MA was reported with a massive DNA damage in many culture cell lines and we interrogated whether 3-MA was damaging the DNA in hDPCs . DNA damage was evaluated by the presence of the phosphorylated histone H2A.X at Ser139, also known as γ-H2A.X ([Rogakou et al., 1998](https://www.frontiersin.org/articles/10.3389/fphar.2020.580343/full#B49))(Chicote et al., 2020). As shown in [Figure S1](https://www.frontiersin.org/articles/10.3389/fphar.2020.580343/full#f9), the levels of γ-H2A.X did not respond to growing concentrations of 3-MA. These results indicated 5mM 3-MA is non-cytotoxic concentrations for hDPCs.


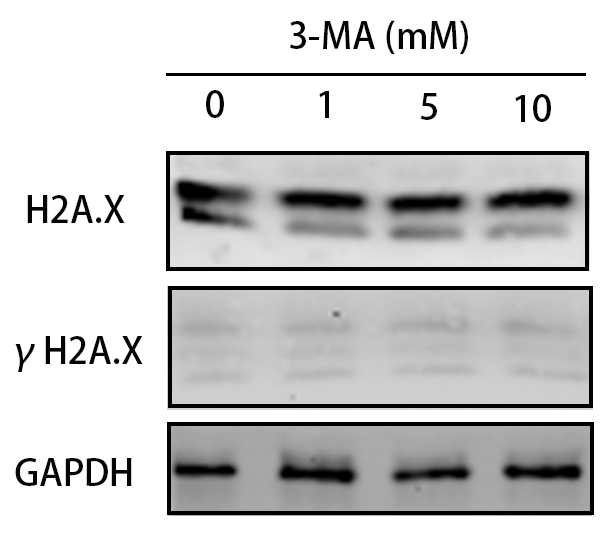


15kDa

36kDa

15kDa

Figure S1. Western blots of the phosphorylated Ser139-H2A.X (γ-H2A.X) and total H2A.X are shown in the hDPCs treated with 3-MA at 0, 1, 5, and 10 mM for 24 h.

References:

Chicote, J., Yuste, V.J., Boix, J., and Ribas, J. (2020). Cell Death Triggered by the Autophagy Inhibitory Drug 3-Methyladenine in Growing Conditions Proceeds With DNA Damage. Front. Pharmacol. 11, 580343. doi: 10.3389/fphar.2020.580343

Rogakou EP, Pilch DR, Orr AH, Ivanova VS, Bonner WM. (1998). DNA double-stranded breaks induce histone H2AX phosphorylation on serine 139. J Biol Chem. 273,5858-68. doi: 10.1074/jbc.273.10.5858.
